# Synergistic Effects of *Vitis vinifera* L. and *Centella asiatica* against CCl_4_-Induced Liver Injury in Mice

**DOI:** 10.3390/ijms241411255

**Published:** 2023-07-09

**Authors:** Suvesh Munakarmi, Yamuna Gurau, Juna Shrestha, Prabodh Risal, Ho Sung Park, Geum-Hwa Lee, Yeon Jun Jeong

**Affiliations:** 1 Research Institute of Clinical Medicine of Jeonbuk National University, Biomedical Research Institute, Jeonbuk National University Hospital, Jeonju 54907, Republic of Korea; sanghzain@gmail.com (S.M.); yamangurau@gmail.com (Y.G.); hspark@jbnu.ac.kr (H.S.P.); 2 Alka Hospital Private Limited, Jwalakhel, Kathmandu 446010, Nepal; zhun12an@gmail.com; 3 Department of Biochemistry, School of Medical Sciences, Kathmandu University, Dhulikhel 45200, Nepal; prabodhrisal@kusms.edu.np; 4 Department of Pathology, Jeonbuk National University Hospital, Jeonju 54907, Republic of Korea; 5 Department of Pharmacology and New Drug Development Research Institute, Jeonbuk National Hospital, Jeonju 54907, Republic of Korea; vitamin2635@naver.com; 6 Department of Surgery, Jeonbuk National University Hospital, Jeonju 54907, Republic of Korea

**Keywords:** VCEC, CCl_4_, oxidative stress, inflammation, antioxidant

## Abstract

Liver injury can be acute or chronic, resulting from a variety of factors, including viral hepatitis, drug overdose, idiosyncratic drug reaction, or toxins, while the progression of pathogenesis in the liver rises due to the involvement of numerous cytokines and growth factor mediators. Thus, the identification of more effective biomarker-based active phytochemicals isolated from medicinal plants is a promising strategy to protect against CCl_4_-induced liver injury. *Vitis vinifera* L. (VE) and *Centella asiatica* (CE) are well-known medicinal plants that possess anti-inflammatory and antioxidant properties. However, synergism between the two has not previously been studied. Here, we investigated the synergistic effects of a *V. vinifera* L. (VE) leaf, *C. asiatica* (CE) extract combination (VCEC) against CCl_4_-induced liver injury. Acute liver injury was induced by a single intraperitoneal administration of CCl_4_ (1 mL/kg). VCEC was administered orally for three consecutive days at various concentrations (100 and 200 mg/kg) prior to CCl_4_ injection. The extent of liver injury and the protective effects of VCEC were evaluated by biochemical analysis and histopathological studies. Oxidative stress was evaluated by measuring malondialdehyde (MDA) and glutathione (GSH) levels and Western blotting. VCEC treatment significantly reduced serum transaminase levels (AST and ALT), tumor necrosis factor-α (TNF-α), and reactive oxygen species (ROS). CCl_4_- induced apoptosis was inhibited by VCEC treatment by reducing cleaved caspase-3 and Bcl2-associated X protein (Bax). VCEC-treated mice significantly restored cytochrome P450 2E1, nuclear factor erythroid 2-related factor 2 (Nrf2), and heme oxygenase-1 (HO-1) expression in CCl4-treated mice. In addition, VCEC downregulated overexpression of proinflammatory cytokines and hepatic nuclear factor kappa B (NF-κB) and inhibited CCl4-mediated apoptosis. Collectively, VCEC exhibited synergistic protective effects against liver injury through its antioxidant, anti-inflammatory, and antiapoptotic ability against oxidative stress, inflammation, and apoptosis. Therefore, VCEC appears promising as a potential therapeutic agent for CCl_4_-induced acute liver injury in mice.

## 1. Introduction

The liver is a vital organ that plays a crucial role in performing various bodily functions such as storage, production, metabolism, and detoxification of harmful substances within the body [1,2]. Nevertheless, the oxidative stress and lipid peroxidation that occur as a result of repeated exposure to toxic chemicals promotes liver damage [3]. Among these chemicals, carbon tetrachloride (CCl_4_) is a highly toxic compound widely used in experimental research to study liver injury [4]. In the liver, cytochrome P450 enzymes convert CCl_4_ into free radicals such as trichloromethyl radical (•CCl_3_) and peroxy trichloromethyl radical (•OOCCl_3_), which are responsible for the induction of oxidative stress, inflammation, and cellular damage [4]. Thus, a better understanding of this mechanism will be useful in developing potential therapeutic strategies to prevent or treat CCl_4_-induced liver injury.

Oxidative stress and inflammation are two major pathophysiological mechanisms that play a crucial role in the progression of liver injury by triggering the production of reactive oxygen species (ROS) and the antioxidant defense system [4,5,6,7]. The liver has a variety of antioxidant defense mechanisms, including both enzymatic and nonenzymatic antioxidants such as superoxide dismutase (SOD), catalase (CAT), glutathione peroxidase (GPx), and glutathione (GSH), that help to maintain the proper balance against oxidative stress and protect the liver from damage [8,9,10]. Therefore, interventions that target both oxidative stress and inflammation may be effective in preventing or treating acute liver injury.

Current treatments for liver disease with conventional and newer synthetic drugs can be challenging due to poor management and serious side effects. Thus, a need exists to explore traditional and herbal medicines as potential therapies for liver disease is required [2]. *Vitis vinifera* L. (VE) is a species of grapevine plant used mainly for herbal medicine as a food supplement, whereas *Centella asiatica* L. (CE), also known as Gotu Kola, is a herbaceous plant that has been used in traditional medicine to treat skin conditions, respiratory problems, and anxiety [11,12]. Both VE and CE possess antioxidant and anti-inflammatory properties and have been studied for their prophylactic effects against various diseases [13,14]. Earlier studies have shown that VE is hepatoprotective against CCl_4_-induced acute liver injury and alcohol-induced oxidative damage in rats [11,15]. The synergistic effects of these phytochemicals have generated increasing interest as investigators aim to develop more effective therapies for treating various diseases, including cancer, cardiovascular disease, diabetes, and neurodegenerative disorders. However, the protective mechanism behind combinatorial effects is likely to be complex and multifactorial, and further research is needed to fully understand how different protective agents can work together to enhance their overall protective effect. Here, we investigate the synergistic effects of a standardized herbal combination of VE and CE extract against CCl_4_-induced acute liver injury in mice.

## 2. Results

### 2.1. VE and CE in Combination Acted Synergistically and Demonstrated the Highest Antioxidant Activities

**Figure 1:**
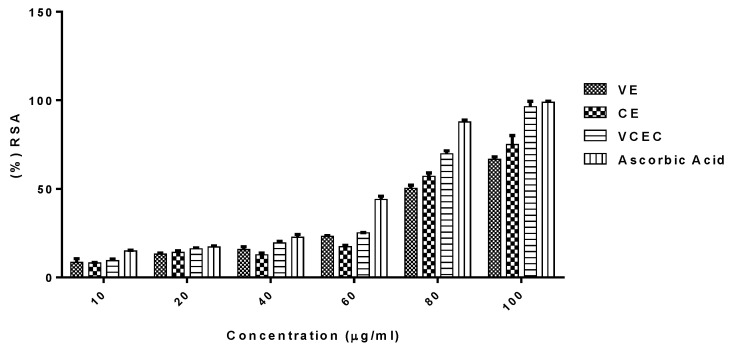
Potential antioxidant properties of VE, CE, and VCEC in comparison with vitamin C. The antioxidant activities of VE, CE, and VCEC were evaluated by DPPH radical scavenging assay.

The antioxidant properties of VE, CE, and VCEC were determined by DPPH (2,2-diphenyl-1-picrylhydrazyl) radical scavenging activity. As shown in Figure 1, VCEC exhibited notably more antioxidant activity than VE and CE alone in concentration-dependent order. VCEC exhibited a level of radical-scavenging activity (RSA) that was comparable to vitamin C. Hence, VCEC demonstrated greater antioxidant properties than either VE or CE alone and did so synergistically.

### 2.2. VCEC ameliorates CCl_4_-Induced Liver Injury

**Figure 2:**
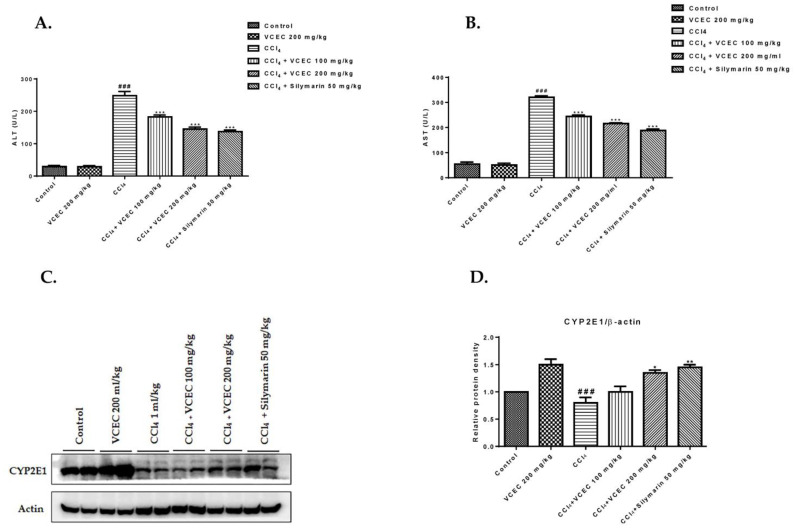
VCEC pretreatment ameliorates CCl_4_-induced acute liver injury in mice. (**A**) Serum levels of alanine aminotransferase (ALT) and (**B**) aspartate aminotransferase (AST) were analyzed 24 h after CCl_4_ injection. Values are presented as mean ± SD (*n* = 7). (**C**) Protein expression of cytochrome P450 2E1 (CYP2E1) 24 h after CCl_4_ injection. (**D**) Quantification of relative protein expression normalized to β-actin. Data are expressed as mean ± SD *(n* = 3). ### *p* < 0.001 denotes a significant difference compared to the control group; * *p* < 0.05, ** *p* < 0.01, and *** *p* < 0.001 indicates significant differences compared to the CCl4-treated group.

To determine the protective role of VCEC, a mouse model of CCl_4_-induced acute liver injury was established. To determine liver function, serum ALT, AST, and protein expression of CYP2E1 were evaluated, as shown in Figure 2. The results demonstrate that the CCl_4_-treated mice had significantly higher levels of serum ALT and AST and dramatically reduced CYP2E1 expression compared to the control group Figure 2A,B. However, pretreatment with VCEC effectively mitigated the elevation in serum ALT and AST activities and restored CYP2E1 expression Figure 2C. This suggests that VCEC possesses hepatoprotective effects against CCl_4_-induced acute liver injury.

### 2.3. VCEC Reduces Hepatic Histopathological Damage Caused by CCl_4_

**Figure 3:**
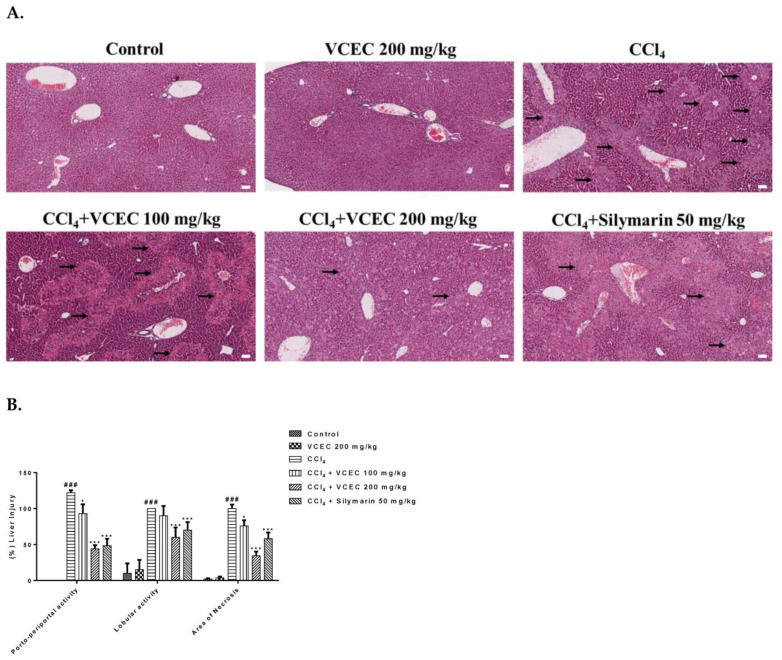
VCEC reduces the histopathological changes caused by CCl_4_ in liver tissues. (**A**) Representative H&E-stained liver sections from the indicated groups with necrotic areas and liver damage as indicated by black arrows. (**B**) Quantitative assessment of (%) area score of liver damage in the indicated groups. The H&E-stained tissues were observed under light microscope at magnification of ×100. The white bar in figure represents the scale bar of 50 µmm, respectively. The values presented are expressed as the mean ± SD (*n* = 7). ### *p* < 0.001 denotes significancy compared with the control group; * *p* < 0.05 and *** *p* < 0.001 indicates significant differences compared with the CCl4-treated group.

To determine the level of histopathological damage caused by CCl_4_ and to evaluate the protective role of VCEC, H&E staining of liver sections was performed, as shown in Figure 3. Histopathology caused by CCl_4_ was manifested by loss of liver structure, hepatocyte necrosis, inflammatory cell infiltration, and increased intercellular gaps and was significantly attenuated by pretreatment with VCEC (100 mg/kg and 200 mg/kg) and silymarin (50 mg/kg), in a dose-dependent manner. The extent of liver injury scoring was performed on the basis of previous studies [16].

### 2.4. Antioxidant Properties of VCEC against CCl_4_-Induced Oxidative Stress

The use of antioxidants to protect against CCl_4_-induced liver injury by reducing lipid peroxidation, inhibiting the production of proinflammatory mediators, and restoring normal antioxidant enzyme levels has gained significant attention in recent years [17,18]. In the present study, we investigated the effects of VCEC on the antioxidant status of the liver.

First, the antioxidant potential of phytochemical extracts on LPS-induced hepatotoxicity was *in*, as shown in Figure S1. AML12 cells were either left untreated or pretreated with various concentrations of individual extracts VE (4.7, 9.4, 18.8, and 37.8 µg/mL), CE (1.6, 3.2, 6.2, and 12.5 µg/mL), and VCEC (2.5, 6.3, 12.5, 37.5, and 50 µg/mL). Following LPS treatment (5 µg/mL), protein levels of antioxidant-related markers were significantly reduced. However, pretreatment with VCEC remarkably increased the expression of antioxidant-related protein markers such as GPx4, Nrf2, and HO-1 in a concentration-dependent manner. VCEC was much more potent than either VE or CE alone, as shown in Figure S1.

**Figure 4:**
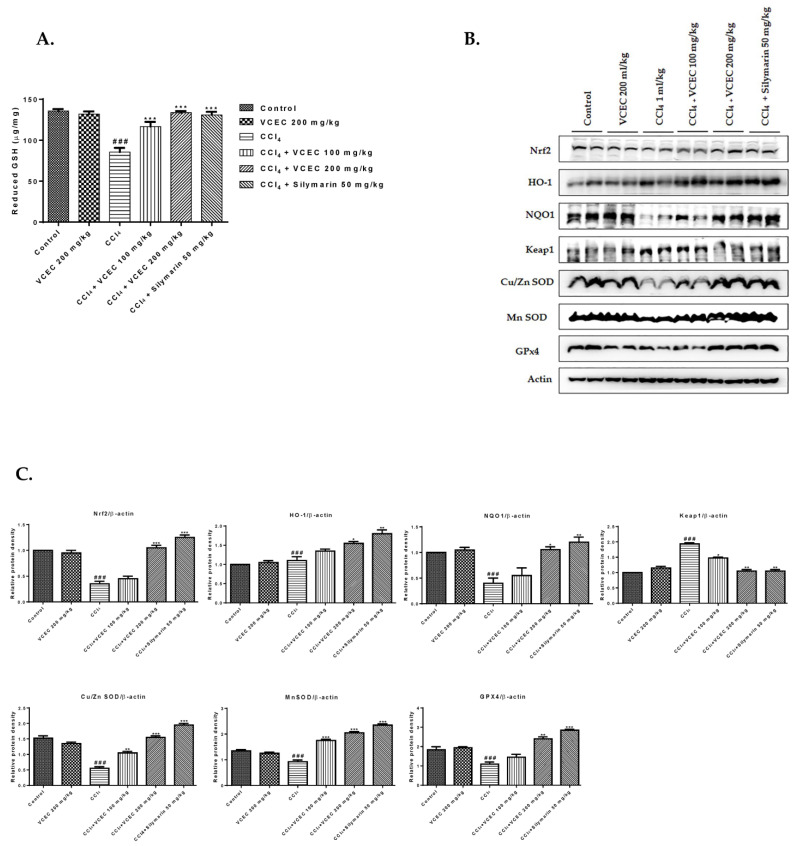
VCEC enhanced antioxidant activity by regulating the Nrf2/HO-1 pathway and inhibiting oxidative stress in CCl_4_-induced acute liver injury in mice. (**A**) Reduced glutathione (GSH) levels. Data presented are expressed as mean ± SD (*n* = 7). (**B**) Western blot analysis determining the protein expression of antioxidant-related markers GPx4, Nrf2, HO-1, and Cu/Zn SOD. (**C**) Quantification of relative protein expression normalized to β-actin. The values presented are expressed as mean ± SD (*n* = 3). ### *p* < 0.001 denotes significancy compared with the control group; * *p* < 0.05, ** *p* < 0.01, and *** *p* < 0.001 indicates significant differences compared with the CCl4-treated group.

On the basis of preliminary studies, we next established a mouse model of CCl_4_-induced acute liver injury to investigate the antioxidant mechanism of VCEC. As shown in Figure 4A, pretreatment with VCEC significantly improved the endogenous levels of reduced GSH. Additionally, Western blot analysis was used to measure the levels of GPx4, Cu/Zn SOD, Nrf2, and its target genes, as shown in Figure 4B, VCEC pretreatment significantly restored protein levels of Nrf2, HO-1, GPx4, and Cu/Zn SOD in compared with the CCl_4_ only treated group. These results suggest that the protective effects of VCEC against CCl_4_-induced oxidative injury are mediated by enhancing antioxidant defense mechanisms through Nrf2/HO-1 activation and reducing oxidative stress.

### 2.5. VCEC Alleviated Lipid Peroxidation, Inflammation, and ROS Production in Response to CCl_4_ Treatment

Measuring inflammatory responses, ROS production, and lipid peroxidation, is important for defining the extent of pathogenesis related to liver injury. Therefore, we first explored the effects of VCEC on ROS production and inflammatory responses. As shown in Figures S2 and S3, LPS treatment dramatically upregulated the levels of intracellular ROS and inflammatory cytokines in AML12 cells; however, VCEC pretreatment inhibited the overproduction of ROS and reduced the expression of inflammatory markers compared to either VE or CE alone.

**Figure 5:**
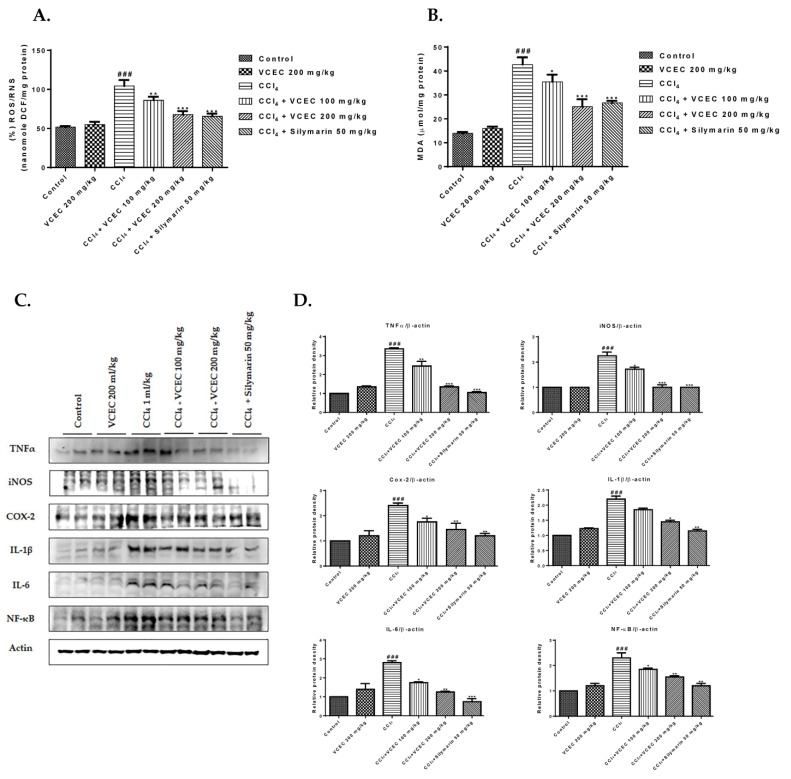
Effects of VCEC extracts on levels of MDA, ROS/RNS, and protein expression level of inflammatory cytokine in CCl_4_-induced acute liver injury. (**A**) Lipid peroxidation as determined by MDA content; (**B**) ROS/RNS level. Data presented are expressed as mean ± SD (*n* = 7). (**C**) Protein expression level of inflammatory cytokines. (**D**) Quantification of relative protein expression normalized to β-actin. Data are expressed as mean ± SD (*n* = 3). ### *p* < 0.001 denotes significancy compared with the control group; * *p* < 0.05, ** *p* < 0.01, and *** *p* < 0.001 indicates significant differences compared with the CCl4-treated group.

Furthermore, we evaluated the protective effects of VCEC against CCl_4_-induced acute liver injury in an in vivo model. Specifically, we measured malondialdehyde (MDA), ROS/RNS levels, and inflammatory cytokine protein expression. As shown in Figure 5A,B, VCEC significantly attenuated the levels of MDA and ROS/RNS in liver tissues compared with the CCl_4_-only-treated group. Similarly, activities of inflammatory cytokines were increased after CCl_4_ treatment; however, pretreatment with VCEC significantly reduced these (Figure 5C).

### 2.6. VCEC Inhibits CCl_4_-Induced Hepatocyte Apoptosis in Mice

**Figure 6:**
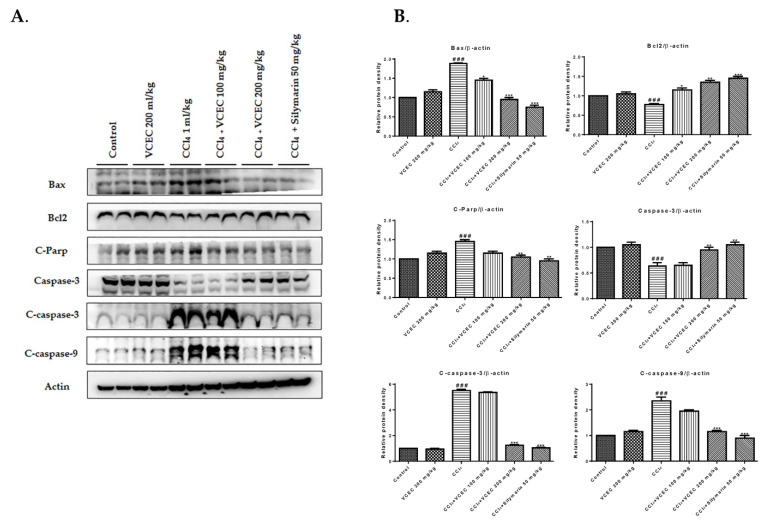
VCEC pretreatment ameliorates the CCl_4_-induced apoptotic response. (**A**) Immunoblot analyses showing the levels of apoptotic proteins (cleaved caspsase-3, 9, and cleaved parp), proapoptotic protein Bax, and antiapoptotic protein Bcl2. (**B**) Quantification of relative protein expression normalized to β-actin. The values represent the mean ± SD (*n* = 3). ### *p* < 0.001 denotes significancy compared with the control group; * *p* < 0.05, ** *p* < 0.01, and *** *p* < 0.001 indicates significant differences compared with the CCl4-treated group.

Apoptosis serves as a mechanism to eliminate damaged or injured hepatocytes, thus contributing to the progression of liver injury [19]. To evaluate the hepatoprotective effect of VCEC against CCl_4_-induced apoptosis, we performed immunoblotting. The results, as depicted in Figure 6, demonstrate that CCl_4_ administration led to a significant decrease in the expression of the antiapoptotic protein Bcl2, along with an increase in the expression of the proapoptotic protein Bax. However, pretreatment with VCEC reversed these effects by restoring Bcl2 levels and reducing Bax. Additionally, VCEC pretreatment reduced CCl_4_-induced apoptotic responses by ameliorating the expression of cleaved caspase-3, 9, and parp compared with the CCl_4_-only group. These findings suggest that VCEC exerts a protective effect against CCl_4_-induced liver injury by suppressing the apoptotic pathway.

## 3. Discussion

The liver plays a pivotal role in various metabolic processes, including detoxification, protein synthesis, and bile production [1]. Damage in the liver results in the impairment of liver function, causing serious health problems. Among several causes, acute liver injury is a drug-induced liver injury caused by exposure to toxins such as CCl_4_. CCl_4_ is a potent liver toxin that can cause severe damage in both animals and humans and is characterized by hepatocellular necrosis, inflammation, and fibrosis [20,21]. Earlier work has shown the core mechanism of CCl_4_ through the formation of highly reactive free radicals that, in turn, cause oxidative stress, lipid peroxidation, and reactive oxygen species generation (ROS) in hepatocytes [22,23]. The extent of liver injury can be evaluated by measuring biochemical markers such as serum ALT and AST, as well as histological analysis of liver tissue [24]. Therefore, in this study, we evaluated the extent of liver damage in our CCl_4_ model by use of various biomarkers, such as serum ALT and AST, the level of MDA as a marker for lipid peroxidation, the carbonyl protein group as an indicator of protein oxidation, and TNF-α as an indicator of inflammation. Collectively, the use of these markers can provide a comprehensive assessment of liver damage and oxidative stress, which can help to shed light on the underlying mechanisms behind liver disease and assist in developing new treatments.

Due to potentially serious side effects, toxicity, drug interactions, and resistance, the use of conventional and synthetic drugs for the treatment of liver disease remains controversial. Therefore, the concept of developing plant-derived natural therapeutics is becoming increasingly popular. CE, also known as Gotu Kola, and VE, commonly known as grapevine, are two herbal medicines with antioxidant and anti-inflammatory qualities that can protect cells from oxidative stress and prevent cellular damage [25,26,27]. There is growing evidence to suggest that different herbs may have complementary or synergistic effects when combined, resulting in more potent and effective treatments [28,29]. Previous studies have noted that the antioxidant capacity of VCEC is substantially higher than that of VE or CE alone [25]. Consistent with other studies, we found that the radical scavenging activity of VCEC was significantly greater and exhibited higher, dose-dependent, free radical scavenging capacity than antioxidant positive control vitamin C and VE or CE alone. Several studies have found that VE and CE have potential hepatoprotective activity against CCl_4_ and ethanol-induced liver toxicity [11,30,31,32,33]. However, more research is needed to fully understand why the combination of the two extracts has synergistic effects in reducing liver damage compared to using either extract alone. In the present study, we attempted to explore the hepatoprotective effects of combining extracts VCEC against CCl_4_-induced acute liver injury in mice and evaluate its ability to enhance antioxidative effects and reduce both inflammation and ROS.

Oxidative stress and inflammation are two important factors that play a critical role in the pathophysiology of CCl_4_-induced liver injury [2,4,34]. Antioxidant response elements (AREs) such as Nrf2 and enzymes such as superoxide dismutase (SOD), MDA, catalase, glutathione peroxidase (GPx), heme oxygenase (HO-1), and glutathione reductase (GR), are crucial in mitigating CCl_4_-induced inflammation in liver injury by scavenging ROS and maintaining the cellular redox balance [35,36]. Accumulating evidence has revealed that parameters such as MDA, SOD, CAT, and reduced glutathione can be used to monitor oxidative stress in mouse models of liver injury. In this study, we showed that VCEC significantly inhibited the production of MDA, restored the levels of reduced GSH, and also enhanced the expression of antioxidant response elements Nrf2 and enzymes HO-1 in the CCl_4_-damaged liver. Furthermore, scavenging DPPH radicals is a major antioxidation mechanism that inhibits the chain reaction leading to lipid peroxidation [37,38]. The results of VCEC DPPH assays indicate that VCEC had a potent radical scavenging capability, which might be helpful in counteracting the pathological changes induced by the free radical CCl_3_, which is induced by CCl_4_. These findings suggest that VCEC effectively protects against CCl_4_-induced hepatic lipid peroxidation by preventing the downregulation of antioxidant enzyme activities, thereby mitigating oxidative damage to the liver.

Oxidative stress can activate inflammatory pathways, while conversely, inflammation can increase oxidative stress by promoting the production of ROS [39,40]. ROS causes oxidative damage, lipid peroxidation, and DNA damage while activating NF-κB signaling and proinflammatory cytokine production [5,41]. Increased oxidative stress and inflammatory mediators, such as TNF-α, IL-1β, and IL-6, are produced as a result of Kupffer cell activation and further stimulate infiltrating macrophages and neutrophils, whereas inflammation is also associated with the production of inflammatory cytokines, iNOS, and COX-2 that contribute to the pathogenesis of liver injury [2,4,42]. The combination of oxidative stress and the release of inflammatory mediators leads to tissue damage, hepatocyte apoptosis, and the development of liver fibrosis [43]. Prior research has revealed that various plant extracts, including VE and CE, have the ability to induce antioxidant enzymes, regulate oxidative stress pathways, and attenuate the production of inflammatory cytokines, leading to liver prophylaxis [25,44,45]. In our study, CCl_4_ administration significantly upregulated the expression of TNF-α, IL-1β, IL-6, iNOS, and COX-2, as well as increased the levels of NF-κB. However, VCEC pretreatment dramatically reduced the upregulation of those proinflammatory cytokines as well as transcription factors, suggesting that VCEC can alleviate CCl_4_-induced liver injury by suppressing the inflammatory response.

Apoptosis and necrosis both contribute to the progression of liver injury [2,4,46]. However, the dominant mode of cell death in CCl_4_-induced liver injury is still unclear [4,47]. Previous studies have reported contradictory findings, with one suggesting necrotic cell death as the primary mechanism [48], while another proposed the involvement of hepatic cell apoptosis [49]. In this study, we focused on the apoptotic pathway, which involves caspase activation. The significant increases we observed in caspases 3 and 9 indicated induction of apoptosis in response to the CCl_4_ administration. However, pretreatment with VCEC downregulated caspase expression, indicating its antiapoptotic activity. Furthermore, apoptosis has been associated with ROS production in mitochondria. Various proapoptotic and antiapoptotic proteins, such as Bax and Bcl2, play a role in cellular apoptosis [50,51]. In the present study, VCEC reversed CCl_4_-induced Bax expression and restored the antiapoptotic protein Bcl2. This is the first time coadministration of these two potent natural extracts has shown a synergistic effect on the apoptosis pathway induced by CCl_4_ in mice. These findings provide valuable insights into the therapeutic potential of VCEC for the treatment or prevention of liver injury. 

In summary, our study demonstrated that the combination of VE and CE extracts had synergistic effects in protecting the liver against CCl_4_-induced ROS, oxidative stress, and inflammation. Compared with VE or CE alone, VCEC exhibits stronger antioxidant and anti-inflammatory properties, making it a promising approach for managing liver diseases. However, additional research is needed to investigate the absorption mechanisms of VCEC and its bioavailability. Understanding how VCEC is absorbed in the body and finding strategies to increase its availability will contribute to optimizing its therapeutic potential against CCl_4_-induced liver injury.

## 4. Materials and Methods

### 4.1. Chemicals and Reagents

CCl_4_ was purchased from Sigma-Aldrich Chemical Co., St. Louis, MO, USA, while serum alanine transaminase (ALT, Cat. # AM 102- K) and aspartate transaminase (AST, Cat. # AM 103-K) assay kits were obtained from Asam Pharm. Co. Ltd., South Korea. Reduced glutathione (GSH, Cat. # DIGT-250) assay kits were purchased from BioAssay Systems, Hayward, CA, USA, while malondialdehyde (MDA Cat. # STA-330) and In vitro ROS/RNS (Cat. # STA-347) assay kits were purchased from Cell Biolabs Inc., San Diego, CA, USA.

### 4.2. Preparation of V. vinifera L. leaf and C. asiatica Extracts 

The *V*. *vinifera* L. leaf (VE) and *C. asiatica* (CE) extracts were obtained from the herbal market in Danyan, China. The extracts were authenticated by the Korea Institute of Oriental Medicine and were prepared as described previously [25,52]. Each extract was mixed at a ratio of 1:3 (1 part of VE and 3 part of CE) to prepare the combination drug VCEC.

### 4.3. DDPH Radical Scavenging Assays

The free radical scavenging activities of VE, CE, and VCEC were evaluated using 2,2-diphenyl-1-picryl-hydrazyl (DDPH). The method was performed as described previously [53]. Briefly, the extracts (2.5 mL) at various concentrations (10, 20, 40, 60, 80, and 100 µg/mL) were added to 1 mL of freshly prepared 0.3 mM alcoholic solution of DDPH. Similarly, standard ascorbic acid solutions of various concentrations were prepared using the same procedure. Control solutions were prepared by replacing the extract with ethanol and 1 mL of DDPH solution. A blank solution was prepared with 3 mL of 95% ethanol. The tube containing the blank, control, and sample was mixed vigorously and allowed to incubate for 30 min in the dark at room temperature. Changes in the absorbance were measured at 518 nm using a spectrophotometer. The percentage of DDPH scavenging activity (% SA) was calculated using the following equation:

(% SA) = (A_0_ − A_1_/A_0_) × 100,

where % SA = percentage scavenging activity;

A_0_ = absorbance of control;

A_1_ = absorbance of sample.

### 4.4. Animals

Specific pathogen-free male C57BL6 mice weighing 20 ± 2 g and aged 7 ~ 8 weeks were purchased from Koatech, Pyeongtake, Korea. Throughout the experiment, mice were provided with a standard diet and had access to water ad libitum. This research adhered to ethical guidelines, and the experimental protocols were approved by the Institutional Animal Care and Use Committee (IACUC) of Jeonbuk National University, Jeonju, South Korea, Approval no: JBUH-IACUC-2023-2.

### 4.5. Animal Model and Drug Treatment

**Figure 7:**
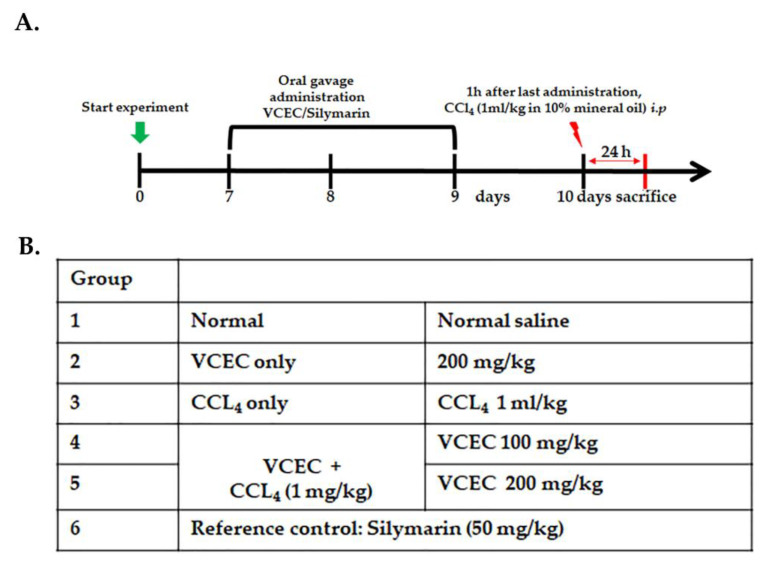
An experimental procedure involved the establishment of a CCl_4_-induced acute liver injury model and subsequent treatment. (**A**) A diagram illustrates the drug administration route and time points at which the animals were sacrificed following CCl_4_ administration. (**B**) A table outlining the different groups along with their corresponding doses of CCl_4_.

A total of 42 mice were divided into six groups, with each group consisting of seven mice. In the control group (Group I) and negative control group (Group III), mice were treated intraperitoneally with normal saline for three consecutive days. Group II mice received an oral administration of 200 mg/kg VCEC only, while Group VI served as the positive control group and received oral administration of 50 mg/kg silymarin for three days. Groups IV and V, known as the CCl_4_ + VCEC groups, were orally administered VCEC at doses of 100 and 200 mg/kg for three consecutive days. On the third day, one hour after the final administration of VCEC, all groups except Groups I and II were injected with a single dose of CCl_4_ (1mg/mL in 10% mineral oil) intraperitoneally. At 24 h after the CCl_4_ injection, mice from each group were anesthetized, blood samples were collected, and then they were sacrificed. Serum from blood and liver tissues were collected from the mice for further analysis. The experimental timeline and details are noted in Figure 7.

### 4.6. Determination of Biochemical Parameters

After blood samples were collected, serum was obtained as described previously [2,4]. The enzymatic activities of serum alanine transaminase (ALT) and aspartate aminotransferase (AST) were determined using commercially available detection kits in accordance with the manufacturer’s instructions. 

### 4.7. Liver Histopathological Examination

Liver tissues were fixed in a 4% paraformaldehyde solution and embedded in paraffin. Tissue sections of 5 µm thickness were prepared and subsequently deparaffinized using xylene. The sections were stained with hematoxylin and eosin (H&E) and observed under a light microscope at a magnification of 100× to determine the tissue morphology. To quantify the degree of liver injury, five randomly selected areas were analyzed using image analysis software. The software enabled the assessment of necrotic foci in liver tissue.

### 4.8. Measurements of Reduced Glutathione (GSH) and Malondialdehyde (MDA)

To determine the amount of reduced GSH and MDA, liver tissues were homogenized and centrifuged at 12,000 rpm for 15 min at 4 °C. Then the resultant supernatant was collected to measure the levels of reduced GSH and MDA using commercially available assay kits from BioAssay Systems (Hayward, CA, USA) and Cell Biolabs, Inc. (San Diego, CA, USA), respectively, following the manufacturer’s instructions. The measurements of reduced GSH were performed spectrophotometrically at a wavelength of 405 nm, while the amount of MDA was determined by measuring the absorbance of the colored complex at a wavelength of 532 nm by kinetic spectrophotometer.

### 4.9. Determination of ROS Formation

In order to assess the intracellular levels of reactive oxygen species (ROS) in AML12 cells, the DCFH-DA staining technique was used. After treatment with desired drugs for 24-h, the cells were exposed to serum-free media containing 10 µmol/L DCFH-DA for 45 min at 37 °C. The specific absorbance of the fluorescent product DCF was quantified using flow cytometry, following the instructions provided by the manufacturer. Furthermore, OxiSelect^TM^ In Vitro ROS/RNS assay kit Cell Biolabs, Inc. (San Diego, CA, USA) was used to quantify the levels of ROS in mice with CCl_4_-induced liver injury. Liver tissue lysates were homogenized and centrifuged at 10,000× *g* for 5 min at 4 °C. The supernatants from homogenized lysates were incubated with DCF-DA solution for 15–45 min at room temperature, and then the fluorescence was observed at 480 nm excitation/530 nm emission wavelengths using a SpectraMax Gemini XS fluorometer as described by manufacturer’s instructions.

### 4.10. Immunoblotting

Cell lysates and liver tissue homogenates were prepared and quantified as described previously [2,4]. Proteins were separated using SDS-PAGE gels and electrotransferred onto PVDF membranes. The membranes were then blocked for 1 h using 5% skim milk and subsequently incubated with specific primary antibodies and horseradish peroxidase-conjugated secondary antibodies. The primary and corresponding secondary antibodies used in this study are listed in Table 1. After incubation with respective primary and secondary antibodies, the protein signals were enhanced and detected using a chemiluminescence detection device.

### 4.11. Statistical Analysis

The findings presented in this study are derived from a minimum of three repeated experiments and are reported as mean values with corresponding standard deviations. Statistical analysis was conducted using GraphPad Prism software (Graph Pad v5 Software, San Diego, CA, USA). The significance of the data was assessed using one-way ANOVA, followed by Tukey’s post hoc test. A *p*-value of less than 0.05 (*p* < 0.05) was considered statistically significant.

## 5. Conclusions

**Figure 8:**
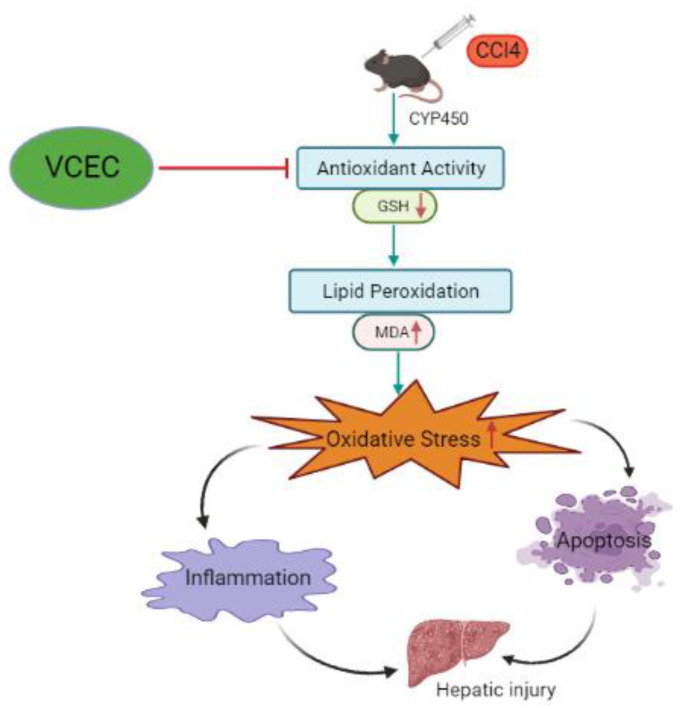
A schematic diagram of the proposed mechanism by which VCEC exerts its effects on CCl_4_-induced acute liver injury through multiple pathways. CCl_4_ exposure leads to a decrease in antioxidant capacity, represented by reduced levels of SOD, GSH, and Nrf2, indicated by downward red arrows. Additionally, CCl_4_ administration increases inflammatory responses, oxidative stress, and apoptotic protein levels, depicted by upward red arrows. However, VCEC pretreatment counteracts these effects.

We evaluated the hepato-protective and synergistic effects of VCEC on CCl_4_-induced acute liver injury in mice and explored the underlying mechanisms. The results indicate that VCEC mitigated CCl_4_-induced damage, as shown by reduced liver histopathology and serum ALT and AST levels. The combined phytochemical extracts that comprise VCEC primarily ameliorate liver injury by inhibiting the NF-κB pathway, with subsequent suppression of inflammatory responses. In addition, VCEC alleviated hepatic oxidative stress by activating antioxidant response elements. These distinct mechanisms of action highlight the potential of VCEC as a therapeutic agent for liver injury and suggest that the effects may be attributed to different pathways that target inflammation and oxidative stress, respectively (Figure 8).

## Figures and Tables

**Table 1:** List of specific primary and secondary antibodies used for immunoblotting.

S.N	Target	Blocking Solution	Dilution	Secondary	Manufacturer	Catalogue Number
1	β-actin	5% Skim Milk	1:3000	Mouse IgG	Sigma Aldrich	A5441
2	COX-2	5% BSA	1:1000	Rabbit IgG	Cell Signaling	4842S
3	C-Caspase-9	5% BSA	1:1000	Mouse IgG	Cell Signaling	9508S
4	Parp	5% Skim Milk	1:3000	Rabbit IgG	Santa Cruz	Sc-7150
5	Keap-1	5% Skim Milk	1:3000	Mouse IgG	Santa Cruz	Sc-365626
6	Nrf-2	5% BSA	1:1000	Rabbit IgG	Gene Tex	GTX103322
7	NQO1	5% Skim Milk	1:3000	Mouse IgG	Santa Cruz	Sc-32793
8	HO-1	5% Skim Milk	1:3000	Mouse IgG	Santa Cruz	Sc-136960
9	GPX4	5% Skim Milk	1:3000	Mouse IgG	Santa Cruz	Sc-166570
10	Cu/Zn SOD	5% BSA	1:1000	Rabbit IgG	Enzo	ADI-SOD-100
11	Mn SOD	5% BSA	1:3000	Rabbit IgG	Enzo	ADI-SOD-110
12	iNOS	5% BSA	1:1500	Mouse IgG	R&D System	MAB9502
13	NF-κB	5% Skim Milk	1:3000	Rabbit IgG	Santa Cruz	Sc-8008
14	IL-1β	5% Skim Milk	1:3000	Rabbit IgG	Santa Cruz	Sc-7884
15	IL-6	5% BSA	1:1000	Rabbit IgG	Bio world	BS-6419
16	TNF-α	5% Skim Milk	1:3000	Hamster IgG	Santa Cruz	Sc-12744
17	CYP2E1	5% BSA	1:1000	Rabbit IgG	Abcam	ab28146
18	Bax	5% Skim Milk	1:3000	Mouse IgG	Santa Cruz	Sc-7480
19	Bcl2	5% Skim Milk	1:3000	Mouse IgG	Santa Cruz	Sc-7382
20	Caspase-3	5% Skim Milk	1:3000	Rabbit IgG	Santa Cruz	Sc-7148
21	C-Caspase-3	5% BSA	1:1000	Rabbit IgG	Cell Signaling	#9664

## Data Availability

Not applicable.

## Supplementary Materials

The following supporting information can be downloaded at: https://www.mdpi.com/article/10.3390/ijms241411255/s1, Figure S1: VCEC regulates antioxidant activity in LPS-induced hepatoxicity; Figure S2: VCEC protects against CCl_4_-induced ROS production; Figure S3: VCEC protects against LPS-induced inflammation in hepatocytes.
