# Supplementary material for: Synergistic Effects of Vitis vinifera L. and Centella asiatica against CCl4-Induced Liver Injury in Mice
Source: Int J Mol Sci. 2023 Jul 9;24(14):11255. doi: 10.3390/ijms241411255 (PMC10379123; doi:10.3390/ijms241411255)
Supplement: Supplementary file 1 [file ijms-24-11255-s001.zip › ijms-2488450-supplementary.pdf]

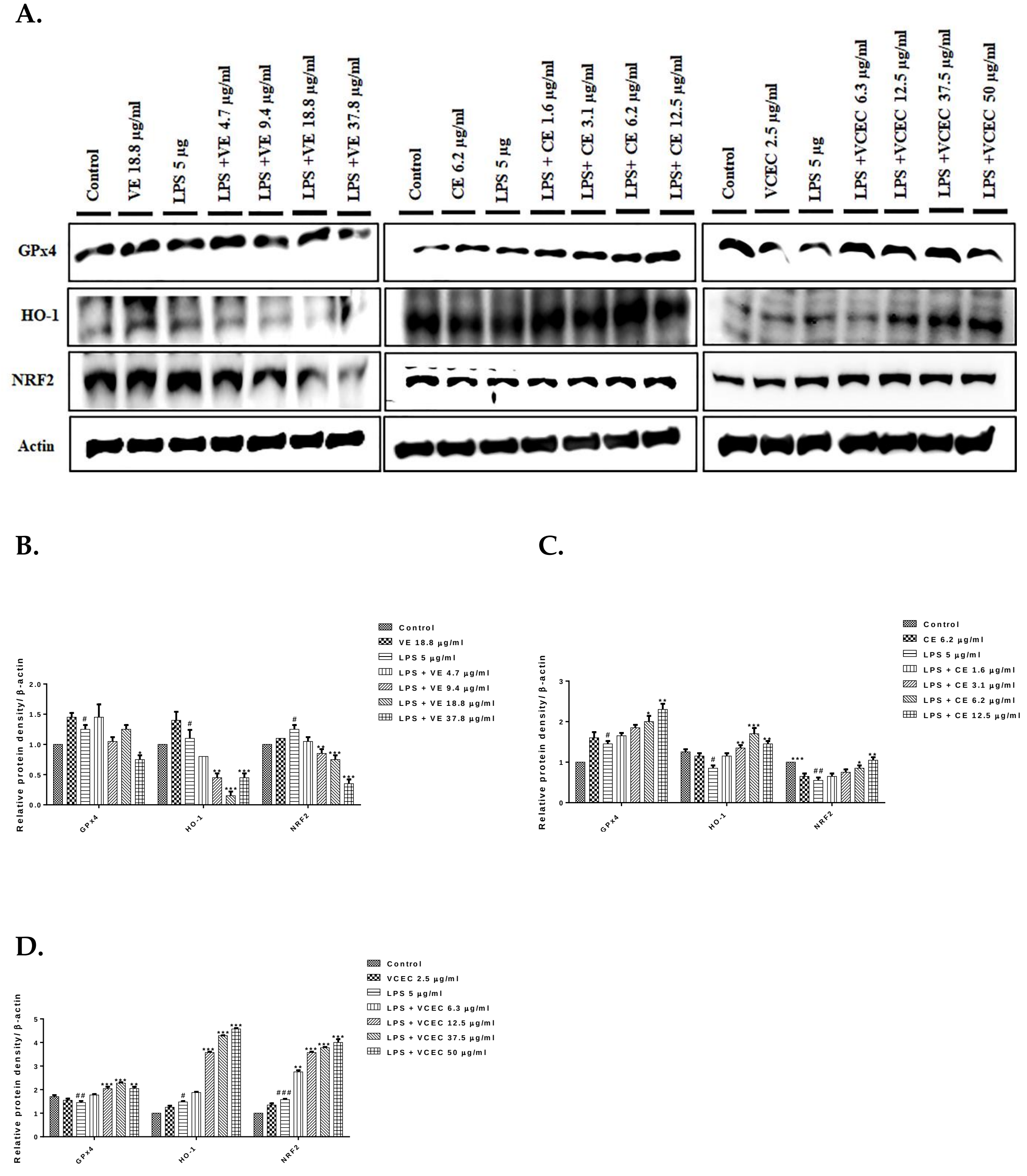

Figure S1. VCEC regulates antioxidant activity in LPS-induced hepatoxicity. As mentioned previously in the methodology section, AML12 cells were treated with individual extracts and LPS at desired concentration for 24 h. (A), Proteins expression antioxidant-related markers were determined by western blotting. (B, C, and D), Quantification of relative protein expression normalized to β-actin. The values represent the mean ± SD (n=3). # $p < 0.05$, ## $p < 0.01$, and ### $p < 0.001$ denotes significancy compared with the control group; * $p < 0.05$, ** $p < 0.01$, and *** $p < 0.001$ indicates significant differences compared with the CCl4-treated group.

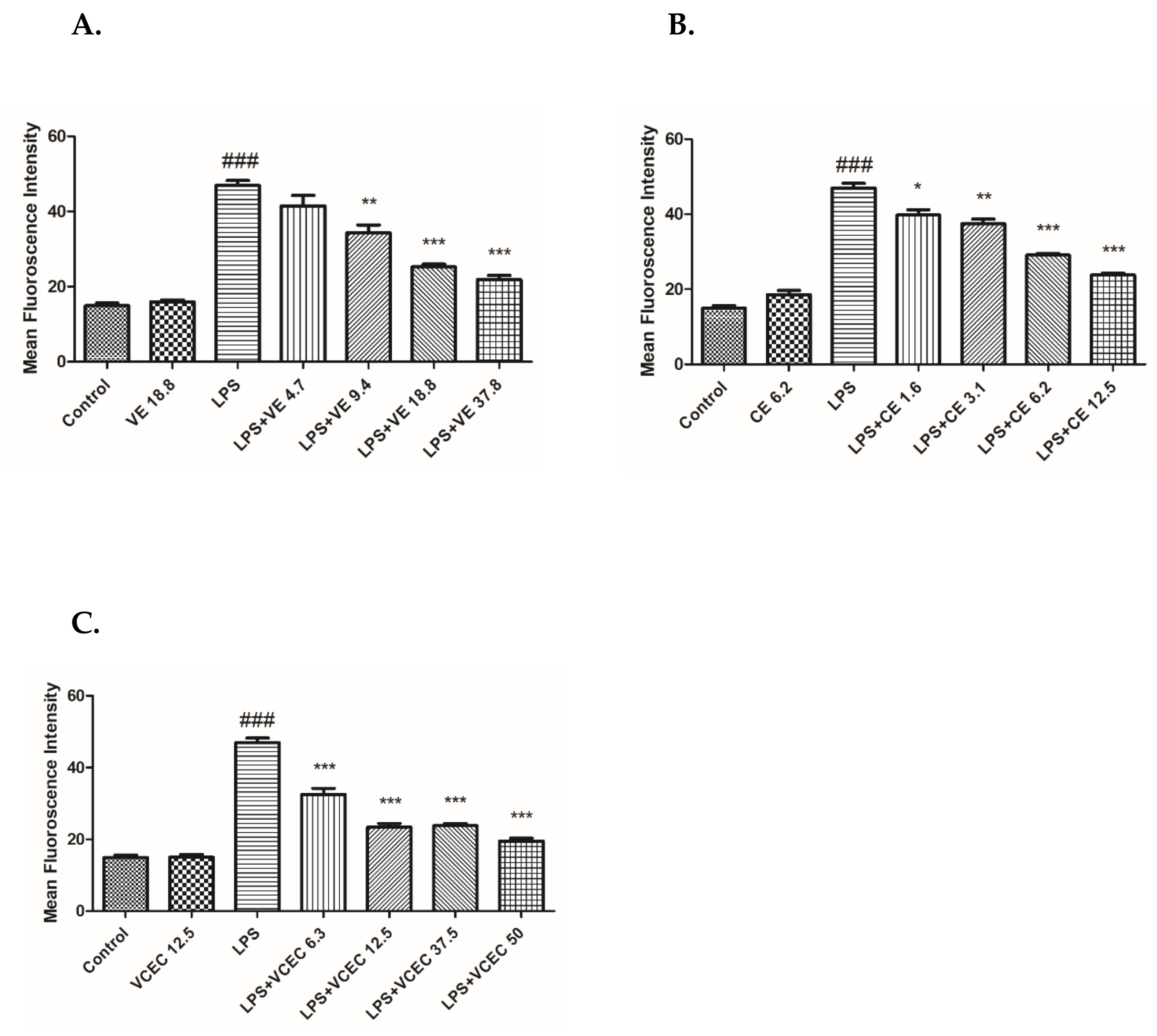

Figure S2. VCEC protects hepatocyte cells from LPS-induced ROS production. (A), (B), and (C) Hepatocyte cells were treated together with 5 μg LPS and desired concentration of individual extracts VE, CE, and VCEC for 24 h. ROS was measured by determining the fluorescence intensity as described in the material and methods section. The values presented are expressed as the mean ± SD (n=7). ### $p < 0.001$ denotes significancy compared with the control group; *** $p < 0.001$ indicates significant differences compared with the CCl4-treated group.

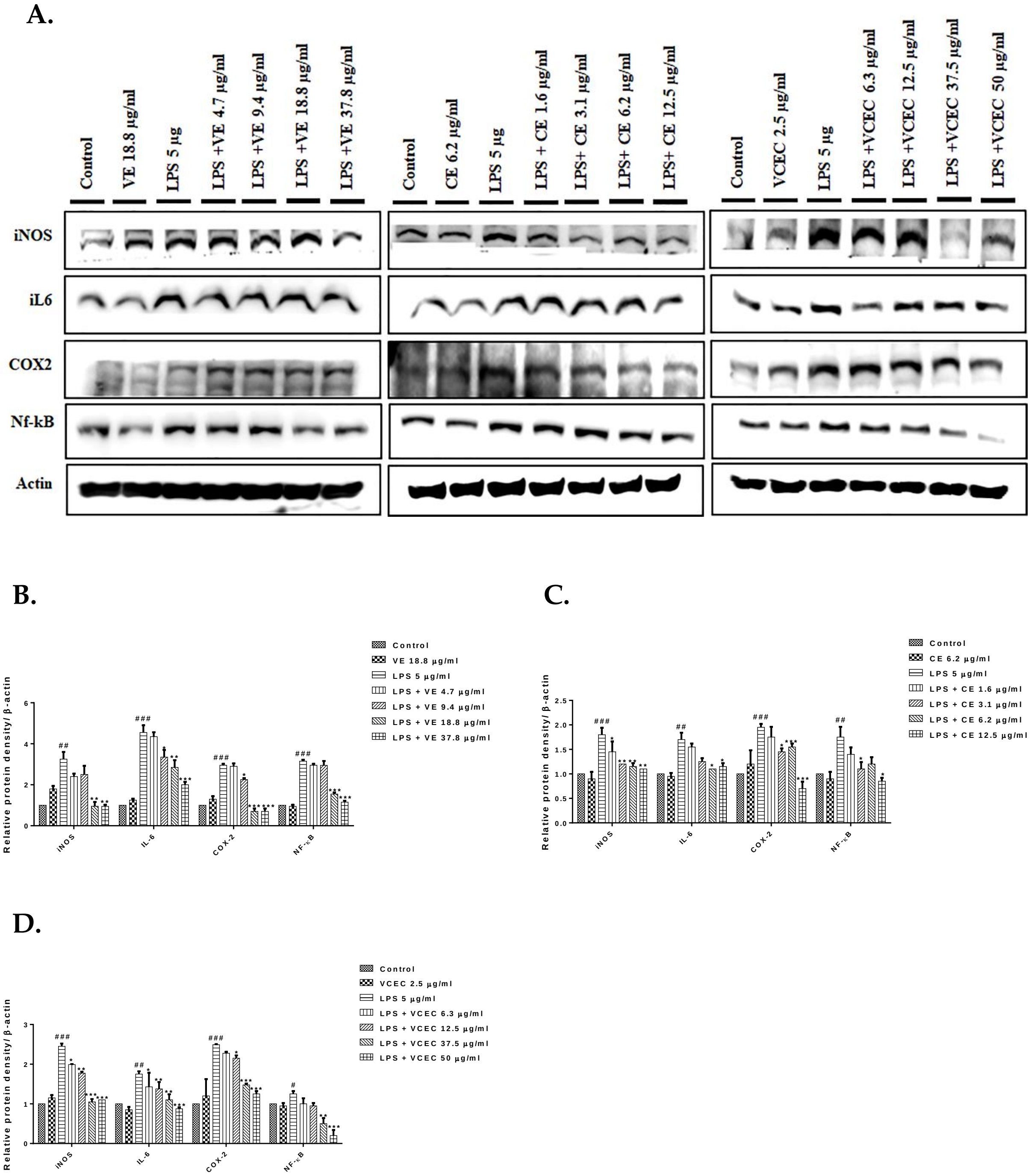

Figure S3. VCEC protects against LPS-induced inflammation in hepatocytes. As mentioned previously in the methodology section, AML12 cells were treated with individual extracts and LPS. (A), Proteins expression of inflammatory cytokines as determined by western blotting. (B, C, and D), Quantification of relative protein expression normalized to β-actin. The values represent the mean ± SD (n=3). # $p < 0.05$, ## $p < 0.01$, and ### $p < 0.001$ denotes significancy compared with the control group; * $p < 0.05$, ** $p < 0.01$, and *** $p < 0.001$ indicates significant differences compared with the CCl4-treated group.
